# Temporal Patterns of Healthcare Provider Referrals in an Emergency Department Research Study

**DOI:** 10.7759/cureus.103481

**Published:** 2026-02-12

**Authors:** Elle (Yueqiao) Wang, Madison Newby, Jessica Moe

**Affiliations:** 1 Emergency Department, University of British Columbia, Vancouver, CAN; 2 School of Nursing, University of British Columbia, Vancouver, CAN; 3 Emergency Department, Vancouver General Hospital, Vancouver, CAN; 4 Emergency Department, BC Children's Hospital, Vancouver, CAN

**Keywords:** buprenorphine/naloxone, clinical research engagement, emergency department, healthcare provider referrals, opioid agonist therapy, temporal patterns

## Abstract

Background: Clinical research in the emergency department (ED) relies on healthcare providers to identify and refer eligible patients, yet the ED’s fast-paced and unpredictable workflow can limit provider capacity for research participation. To inform more efficient engagement strategies for the Evaluating Microdosing in the Emergency Department (EMED) study, which is evaluating methods of initiating buprenorphine/naloxone from EDs, we examined temporal and provider-specific patterns of healthcare provider referrals (HCPRs).

Objective: To quantify temporal variation in referral activity by comparing referral rates per hour across months, days of the week, and work shifts, adjusting for research assistant availability.

Methods: We conducted a retrospective analysis of HCPRs to the EMED study at the Vancouver General Hospital's ED from July 23, 2021, to December 31, 2023. Referral records captured provider type and referral timing and were analyzed during periods of research assistant coverage (morning 07:00-14:59, evening 15:00-22:59, and occasional overnight 23:00-06:59). We calculated referral frequency density (referrals per research-assistant hour), and compared rates across months, days of the week, and shifts using Poisson regression with an offset for research-assistant availability.

Results: From July 2021 to December 2023, we received 2,244 HCPRs, primarily from registered nurses (1,928; 85.9%), followed by physicians (230; 10.2%), other providers (56; 2.5%), pharmacists (26; 1.2%), and social workers (4; 0.2%). After adjusting for research assistant availability, referral rates varied by month (compared to January, higher in December (incidence rate ratio (IRR) = 1.38); lower in September (IRR = 0.77)), by day of the week (compared to Monday, higher on Thursday (IRR = 1.39) and Friday (IRR = 1.20)), and by shift (compared to evenings, higher in mornings (IRR = 1.29); lower in overnights (IRR = 0.37)). Across all months, nurses consistently had the highest referral frequency density, with an apparent increase beginning in October 2022 that coincided with enhanced engagement activities, and a temporary decrease in November 2023 during a brief pause in EMED activity. Referral activity was most commonly observed during morning shifts for nurses, pharmacists, and other providers, whereas physician referrals shifted over time, with increased overnight referral frequency in later years.

Conclusion: HCPRs to EMED were temporally clustered by month, day of the week, and shift after accounting for research assistant availability, and were predominantly contributed by registered nurses, with an apparent increase following enhanced engagement activities. These patterns may help inform the timing and tailoring of future engagement approaches, particularly for provider groups with consistently low referral activity.

## Introduction

In clinical research, healthcare providers are essential to the successful implementation and execution of studies, as they are often responsible for assessing eligibility, administering interventions, and collecting data [1]. Research conducted in the emergency department (ED) presents unique challenges due to the fast-paced and unpredictable nature of the environment [2]. Healthcare providers in the ED are often busy, making it challenging to engage them in research efforts. It is crucial to find effective ways to motivate their participation and determine the optimal times to interact with them to minimize disruption to patient care [3].

The Evaluating Microdosing in the Emergency Department (EMED) study compares the effectiveness of buprenorphine/naloxone take-home induction regimens in enabling ED patients to successfully complete the induction period and retain patients on opioid agonist therapy (OAT) [4]. EMED collaborates with multidisciplinary ED healthcare providers to screen and identify patients at risk for harmful opioid use and engage them in care [5]. In line with the 2017 Canadian Association of Emergency Physicians (CAEP) Academic Symposium's recommendations on engaging emergency clinicians in ED clinical research [6], we have implemented quality improvement strategies to enhance ED provider engagement in EMED. With our ultimate aim to increase healthcare provider engagement in EMED, our ongoing objective is to analyze the timing and patterns of referrals to inform strategies to optimize provider involvement. In this study, we examined healthcare provider referral (HCPR) occurrences by provider type, months, days of the week, and work shifts by comparing referral rates per hour across these periods while accounting for differences in research assistant availability.

## Materials and methods

Study setting

The EMED study has been actively recruiting participants in the ED of Vancouver General Hospital (VGH) since July 2021. Research assistants have regular working shifts from 7:00 AM to 2:59 PM (morning shift) and from 3:00 PM to 10:59 PM (evening shift), seven days a week, excluding statutory holidays. To optimize enrollment, we occasionally coordinate research assistants to cover overnight shifts from 11:00 PM to 6:59 AM. Our study identifies participants primarily via two methods: (1) research assistants conduct broad screening based on the study’s inclusion criteria, and (2) any healthcare provider can refer potentially eligible patients to the study team. We receive referrals through two channels: healthcare providers contact the study research assistants directly (telephone call or text message), and/or healthcare providers document referral-related comments in the electronic medical record, which research assistants could review during broad screening. Once referred, research assistants approach patients to complete assessments for study eligibility.

We collected data on HCPRs during times of research assistant availability. When healthcare providers referred participants to the study, we collected the following information in a standardized study REDCap (Vanderbilt University, Nashville, TN) form: (1) the referral date, (2) the time when research assistants approached and screened the participants, and (3) the type of healthcare provider who made the referral. Referring providers were categorized based on their primary professional role as reported by the referrer: registered nurse, physician, pharmacist, social worker, or other (non-categorized ED staff, including medical students, medical residents, licensed practical nurses, and research staff).

EMED engagement strategy

We implemented a structured engagement strategy to support and sustain ED provider referrals to the EMED study over time. Engagement activities are summarized below; detailed operational procedures and implementation were described in our previously published work [7].

Baseline awareness activities were implemented at the outset of the study and continued throughout the study period. These activities included physical and digital study posters in clinical areas, departmental email updates and newsletters, and informal outreach by research staff during clinical shifts. Beginning in January 2022, we introduced enhanced engagement activities centered on brief, in-person “Coffee Carts” rounds conducted approximately once per month. These sessions consisted of short, approximately five-minute educational interactions delivered by the study team in clinical areas during active shifts, focusing on study awareness, referral processes, and opioid-related clinical education. Content was iteratively adapted based on provider feedback and operational considerations.

In addition to Coffee Carts, the study team actively participated in hospital-led education and knowledge-sharing events for healthcare providers. These included, but were not limited to, education days beginning in June 2022 for nurses and nursing new hires, pharmacists, and physicians. During these sessions, study team members introduced the EMED study, described referral processes, and responded to provider questions.

In 2023, the study team introduced an additional peer-based engagement initiative through a Suboxone Champions program. This program recruited interested physicians, nurses, and pharmacists to serve as peer educators and points of contact for the EMED study, complementing the Coffee Carts initiatives and further supporting ongoing study awareness and referrals.

A brief pause in study enrollment occurred in November 2023 due to operational constraints, after which engagement activities and enrollment resumed.

Statistical analysis

We analyzed HCPR data collected from the beginning of the study (July 23, 2021) to December 31, 2023. To facilitate a robust comparison between these variables, which encompassed different numbers of hours within the specified period, we normalized the HCPR frequency data using a frequency density method.

We computed the frequency density as follows. Frequency (F): the number of HCPRs identified within a specified time frame (e.g., the total number of HCPRs in January 2022). Interval width (W): the total number of hours during which we collected data in the VGH ED for that specific time frame (e.g., the total hours of data collection in January 2022).

We calculated frequency density (D) using the formula: D = F/W.

This normalization process allowed us to standardize HCPR data across varying time intervals, allowing valid and accurate comparisons. Our previous engagement quality improvement research indicated that after we implemented our study-specific engagement strategy in week 25 of the EMED study, referrals showed an improving trend starting from week 52, which corresponds to June 2022 [7]. Given this observed trend, we hypothesized that the referral frequency density would fluctuate over time as a result of our engagement strategy's impact.

To understand how HCPRs varied temporally, we stratified the HCPR frequency density (1) by month and provider type over time, (2) by day of the week and provider type over time, and (3) by shift (morning shift, evening shift, and overnight shift) and provider type over time. We used Poisson regression models with the log of research assistant availability hours included as an offset, and reported effect estimates as incidence rate ratios (IRRs), to compare referral rates per hour among months, days of the week, and work shifts. Statistical significance was assessed using Wald tests, and results were presented as IRRs with 95% confidence intervals (CIs) and two-sided p-values. Months, day of the week, and work shifts were modelled as categorical predictors, with research assistant availability hours included as a log offset to account for differences in exposure across periods. As models were fitted to aggregated counts with only categorical predictors (zero residual degrees of freedom), standard overdispersion diagnostics were not applicable [8]. We did not model an intervention/post-intervention period or any continuous time trend. Referrals with missing or incomplete information on referral time or provider type were retained in the overall counts but were excluded from analyses requiring that specific variable (e.g., shift-based analyses when referral time was missing). Furthermore, we visualized the HCPR frequency density using heatmaps to illustrate patterns across shifts. This approach provided us with a comprehensive understanding of the temporal distribution of HCPRs throughout the study period.

Data cleaning, management, and statistical analyses were performed in Python version 3.10 (Python Software Foundation, Wilmington, Delaware), using the “statsmodels” package for Poisson regression models. Figures were created in Microsoft Excel (LTSC Professional Plus 2021, Microsoft Corporation, Redmond, WA).

## Results

During the study period, we received a total of 2,244 referrals. The majority of referrals came from registered nurses, who contributed 1,928 referrals (85.9%). Physicians followed with 230 referrals (10.2%), other providers contributed 56 referrals (2.5%), pharmacists provided 26 referrals (1.2%), and social workers provided four referrals (0.2%). Other healthcare providers included a small number of non-categorized ED staff, such as medical students, medical residents, licensed practical nurses, and researchers.

We summarized the overall frequency density of HCPRs in Table 1. After adjusting for research assistant availability, monthly referral rates demonstrated significant variation, with higher rates in December (IRR = 1.38, p < 0.001) and lower rates in September (IRR = 0.77, p = 0.02), using January as a baseline comparator. Across days of the week, referral activity was higher on Thursdays (IRR = 1.39, p < 0.0001) and Fridays (IRR = 1.20, p = 0.02) relative to Mondays. We also observed differences by work shift, with higher referral rates during morning shifts (IRR = 1.29, p < 0.0001) and lower rates during overnight shifts (IRR = 0.37, p < 0.0001) compared with evening shifts.

**Table 1:** Overall frequency density of healthcare provider referrals in the EMED study by months, days of the week, and shift type at Vancouver General Hospital ED from study initiation (July 23, 2021) to December 31, 2023 (N = 2,244). * Frequency density: referrals per hour of research assistant availability. ** There were five referrals for which the referral time was not documented. EMED: Evaluating Microdosing in the Emergency Department.

Category	Number of referrals (N = 2,244)	Total number of hours of research assistant availability	Frequency density*	Incidence rate ratio (95% CI)	P-value
Month					
January	156	893.5	0.17	Reference	-
February	151	782.5	0.19	1.11 (0.88 – 1.38)	0.38
March	137	884.5	0.15	0.89 (0.71 – 1.12)	0.31
April	136	814.0	0.17	0.96 (0.76 – 1.20)	0.71
May	153	861.5	0.18	1.02 (0.81 – 1.27)	0.88
June	173	861.0	0.20	1.15 (0.93 – 1.43)	0.20
July	210	992.0	0.21	1.21 (0.99 – 1.49)	0.07
August	194	1,259.0	0.15	0.88 (0.71 – 1.09)	0.25
September	163	1,212.0	0.13	0.77 (0.62 – 0.96)	0.02
October	246	1,284.0	0.19	1.10 (0.90 – 1.34)	0.36
November	207	1,387.0	0.15	0.85 (0.69 – 1.05)	0.14
December	318	1,317.5	0.24	1.38 (1.14 – 1.67)	<0.001
Days of the week					
Monday	284	1,792.0	0.16	Reference	-
Tuesday	312	1,778.0	0.18	1.11 (0.94 – 1.30)	0.21
Wednesday	322	1,778.0	0.18	1.14 (0.97 – 1.34)	0.10
Thursday	391	1,778.0	0.22	1.39 (1.19 – 1.62)	<0.0001
Friday	338	1,778.0	0.19	1.20 (1.02 – 1.40)	0.02
Saturday	299	1,792.0	0.17	1.05 (0.89 – 1.24)	0.53
Sunday	298	1,792.0	0.17	1.05 (0.89 – 1.23)	0.56
Shifts**					
Evening (15:00-22:59)	1,028	7,005.0	0.15	Reference	-
Overnight (23:00-06:59)	93	1,712.0	0.05	0.37 (0.30 – 0.46)	<0.0001
Morning shift (7:00-14:59)	1,118	5,891.0	0.19	1.29 (1.19 – 1.41)	<0.0001

Frequency of HCPR by month and provider type

**Figure 1:**
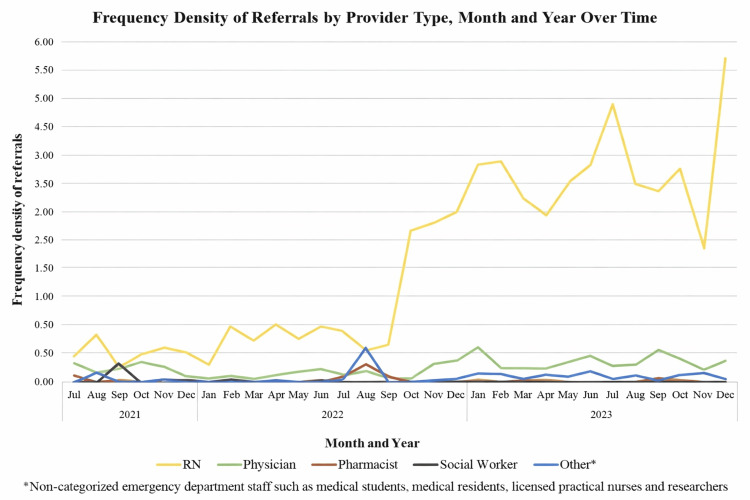
Frequency density of EMED healthcare provider referrals at Vancouver General Hospital by provider type, month, and year from study initiation (July 2021) to December 2023. EMED: Evaluating Microdosing in the Emergency Department; RN: registered nurse.

Over the study period from July 2021 to December 2023, registered nurses consistently had the highest frequency density of referrals in every month (Figure 1). There appeared to be an increasing trend in nursing referral frequency density beginning in October 2022, coinciding with increased engagement activities. Physician referrals remained relatively low and stable over the study period, with slight increases observed around October 2022 and May 2023 (Figure 1). We observed temporary decreases in nursing and physician referrals in November 2023, coinciding with a brief pause in EMED activity. Pharmacist referrals showed low frequency density, with a small increase observed in August 2022, followed by consistently low-level activity thereafter (Figure 1). Social worker referrals remained the lowest among all healthcare providers across all months (Figure 1). Referrals from other provider types were also infrequent, with minor month-to-month fluctuations and no clear overall pattern observed (Figure 1).

Frequency of HCPRs by time of day and provider type over time

**Figure 2:**
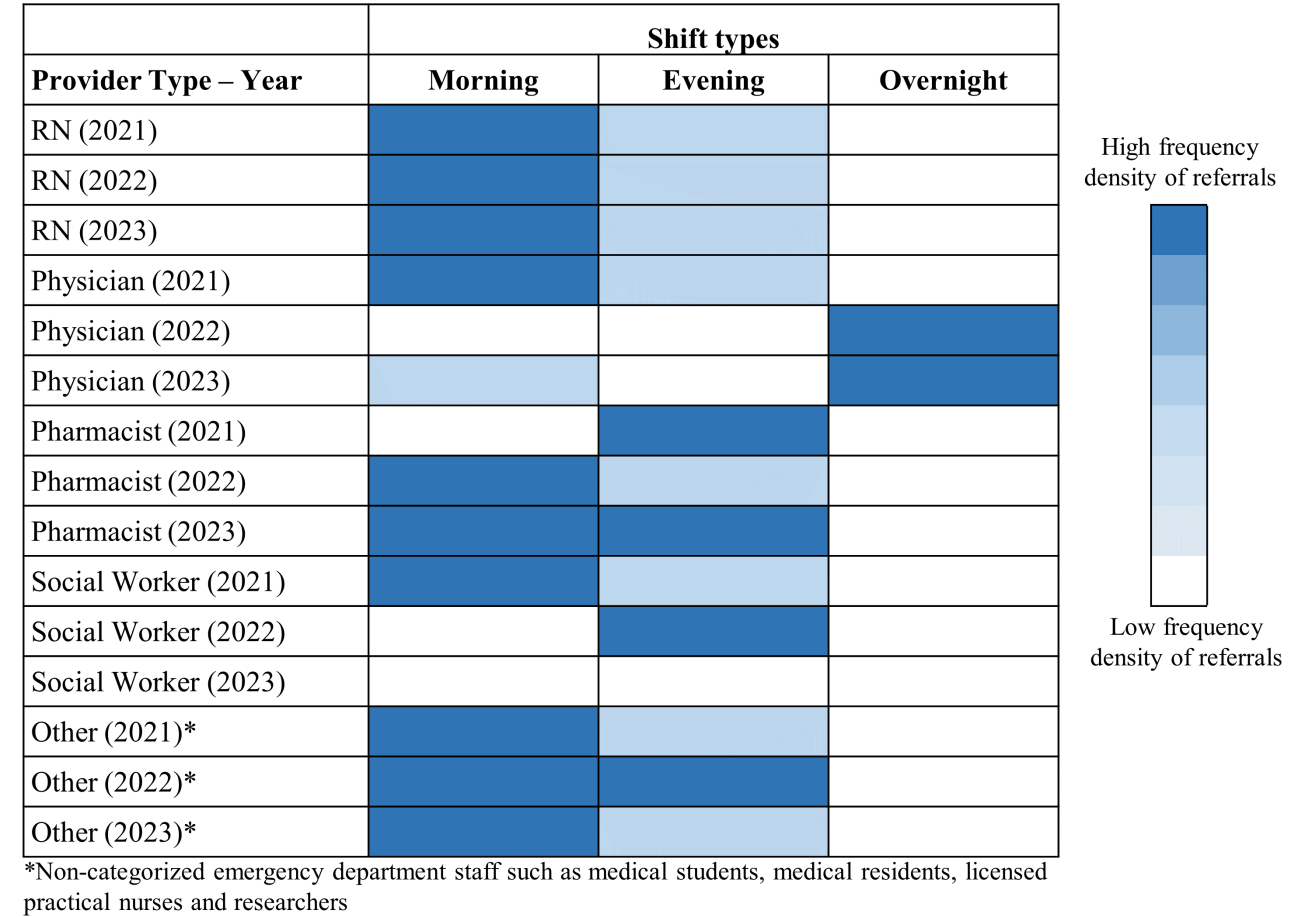
Heatmap of frequency density of healthcare provider referrals at Vancouver General Hospital by shift type, provider type, and year from study initiation (July 2021) to December 2023. RN: registered nurse.

Across provider groups, referral activity was more frequently observed during morning shifts for nurses, pharmacists, and other provider types over the study period (Figure 2). At study initiation, physician referrals occurred most frequently during morning shifts, but during subsequent years, referral frequency increased during overnight shifts (Figure 2).

## Discussion

This study analyzed the patterns of HCPRs over time in the EMED study from July 23, 2021, to December 31, 2023.

We found that HCPRs to the EMED study were not evenly distributed over time. Across the study period (2,244 total referrals), referral frequency density varied by month, day of the week, and shift type after adjusting for research assistant availability. Referral rates were higher in December and lower in September (relative to January), higher on Thursdays and Fridays (relative to Mondays), and highest during morning shifts and lowest during overnight shifts (relative to evening shifts). The reasons underlying this variation were not examined in the present study but warrant further exploration. Improved understanding of why referrals tend to cluster at certain times may help inform more efficient planning of engagement, such as optimizing the timing of awareness activities to align with periods of lower observed referral activity.

When examining referral patterns by provider type across months and years, registered nurses were the dominant contributors, accounting for 85.9% of all referrals. Physicians accounted for 10.2%, while referrals from pharmacists (1.2%) and social workers (0.2%) were uncommon. Registered nurses were the primary contributors to HCPRs: they had the highest frequency density of referrals throughout our study. Physicians and pharmacists showed relatively moderate and stable referral frequencies, and social workers made consistently few referrals over the study period. We observed a general increase in nursing referrals as of October 2022, which likely partially reflects an increase in study engagement activities. Our engagement strategies, such as Coffee Carts and Suboxone Champions programs, primarily targeted nurses, which may explain why temporal increases in HCPRs following engagement activities were mostly observed among nurses. Low referral rates among certain healthcare provider groups suggest an opportunity for targeted interventions to enhance their involvement in research activities. Most referrals from all healthcare providers appeared to occur during morning shifts, although later in our study, we observed that physicians made more referrals during overnight hours.

The differences in HCPR patterns we observed may reflect the varying roles of each provider type in the ED. Registered nurses were the most consistent contributors to HCPRs, likely influenced by their direct involvement in patient care and their constant presence throughout patients’ ED stay. Given their fast-paced duties, strategies in which the research team assists to offload clinical bedside tasks, and engagement activities that minimize disruptions, such as quick, targeted education sessions like Coffee Carts, are essential to maintaining nurses’ participation in research [6,7]. In contrast, physicians face different challenges, such as balancing clinical decision-making, coordinating with consultants, and arranging logistics for patients’ disposition [9], which can limit their time for research activities. Their participation could be enhanced by better integrating research tasks into their clinical workflows and emphasizing the clinical benefits of research [10], as achieved through peer-led initiatives like the Suboxone Champions program [7].

Pharmacists, though fewer in number, play a critical role in research involving medication management [11], such as opioid agonist therapy. Engagement strategies should be tailored to pharmacists’ specialized responsibilities, allowing research tasks to integrate with their routine duties [12]. Social workers contributed the fewest referrals in our study, potentially due to their focus on patient social needs rather than medical interventions [13,14]. Within the ED workflow, potential study patients were typically identified by healthcare providers (e.g., nurses or physicians) or the research team before social workers’ interaction, reducing opportunities for direct referrals. In addition, social work involvement may occur later in the care pathway or may be triggered by specific consult criteria, all of which can limit consistent exposure to the referral process. To increase social workers’ participation in EMED, our team is engaging them to identify ways to integrate their involvement in supporting patients’ socioeconomic needs to enhance the identification of patients with opioid use disorder who may be missed by bedside clinical staff. Evidence suggests that involving social workers earlier in the care continuum can contribute meaningfully to referral processes [15,16].

Our study has limitations. Since registered nurses typically interact with patients first, particularly during triage, a higher frequency of referrals may primarily reflect these initial encounters. Consequently, many eligible patients who might have been identified by physicians, pharmacists, or social workers were likely already referred by the time these providers interacted with the patients. Therefore, our rates of HCPRs may partially reflect ED patient flows rather than provider engagement levels. In addition, referral counts were not adjusted for the underlying number or composition of providers on duty (e.g., nurse-to-physician staffing ratios) across shifts or time periods, which may have contributed to the observed patterns.

We also note that temporal patterns in referrals that coincided with engagement activities should be interpreted as associative rather than causal. In the absence of a formal interrupted time-series or pre/post design, we cannot attribute changes in referral activity to engagement efforts, as other concurrent factors (e.g., operational changes, staffing fluctuations, or seasonal variation in patient presentations) may have influenced referral patterns. Relatedly, our data do not allow us to fully evaluate the underlying drivers of temporal variation in referrals. Finally, the study was conducted at a single site, which may limit the generalizability of the findings to other EDs with different workflows, staffing patterns, and resources. Institutional culture (e.g., departmental norms around research participation and leadership support), staffing models and role delineation (e.g., triage processes, referral pathways, and the availability of pharmacists or social workers across shifts), and differences in patient populations (e.g., case mix and acuity) may influence when and by whom referrals occur; therefore, referral rates and provider-specific patterns may differ across EDs.

However, despite these limitations, our findings offer valuable insights into the engagement of healthcare providers in clinical research within the ED, emphasizing the need for tailored strategies that accommodate the specific needs and constraints of ED healthcare providers to enhance participation. The patterns we observed in nurses’ referral frequencies indicate that intentional engagement strategies can greatly enhance participation in research. Moving forward, our team is seeking to optimize engagement strategies that target healthcare providers with lower referral frequency, particularly at times with lower referral activity.

## Conclusions

This study provides valuable insights into the patterns of HCPRs over time, with variations observed by month, day of the week, and shift type. Registered nurses consistently had the highest referral frequencies, with general increases observed after planned engagement activities. Variations in referral frequencies highlight the need for tailored engagement strategies that account for specific challenges that vary temporally and by provider roles in order to optimize engagement of multidisciplinary ED providers in research.
